# Supplementary material for: Altered microRNA Transcriptome in Cultured Human Liver Cells upon Infection with Ebola Virus
Source: Int J Mol Sci. 2021 Apr 6;22(7):3792. doi: 10.3390/ijms22073792 (PMC8038836; doi:10.3390/ijms22073792)
Supplement: Supplementary file 1 [file ijms-22-03792-s001.zip › Supplementary_File/D_ KEGG_Analysis_Results/16-30nt_pathway_Reston-24h-Huh7_vs_Control-24h-Huh7_down.mature_mirna_targets/hsa_pathwayResult.html]

| PathwayID | Definition | OriginalWebSite | Fisher-Pvalue | SelectionCounts | SelectionSize | Count | Size | FDR | Enrichment\_Score | GeneRatio | Genes |
| --- | --- | --- | --- | --- | --- | --- | --- | --- | --- | --- | --- |
| hsa04978 | Mineral absorption - Homo sapiens (human) | http://www.genome.jp/kegg-bin/show\_pathway?hsa04978+482+486+261729+7421 | 1.865579e-03 | 4 | 79 | 53 | 7906 | 6.231034e-01 | 2.729186 | 0.050633 | ATP1B2//FXYD2//STEAP2//VDR |
| hsa04973 | Carbohydrate digestion and absorption - Homo sapiens (human) | http://www.genome.jp/kegg-bin/show\_pathway?hsa04973+482+486+2542 | 1.135764e-02 | 3 | 79 | 47 | 7906 | 1.000000e+00 | 1.944712 | 0.037975 | ATP1B2//FXYD2//SLC37A4 |
| hsa04961 | Endocrine and other factor-regulated calcium reabsorption - Homo sapiens (human) | http://www.genome.jp/kegg-bin/show\_pathway?hsa04961+482+486+7421 | 1.343907e-02 | 3 | 79 | 50 | 7906 | 1.000000e+00 | 1.871631 | 0.037975 | ATP1B2//FXYD2//VDR |
| hsa04640 | Hematopoietic cell lineage - Homo sapiens (human) | http://www.genome.jp/kegg-bin/show\_pathway?hsa04640+1378+1435+2993+3113 | 1.587447e-02 | 4 | 79 | 97 | 7906 | 1.000000e+00 | 1.799301 | 0.050633 | CR1//CSF1//GYPA//HLA-DPA1 |
| hsa04964 | Proximal tubule bicarbonate reclamation - Homo sapiens (human) | http://www.genome.jp/kegg-bin/show\_pathway?hsa04964+482+486 | 2.177682e-02 | 2 | 79 | 23 | 7906 | 1.000000e+00 | 1.662006 | 0.025316 | ATP1B2//FXYD2 |
| hsa00534 | Glycosaminoglycan biosynthesis - heparan sulfate / heparin - Homo sapiens (human) | http://www.genome.jp/kegg-bin/show\_pathway?hsa00534+9953+64132 | 2.360463e-02 | 2 | 79 | 24 | 7906 | 1.000000e+00 | 1.627003 | 0.025316 | HS3ST3B1//XYLT2 |
| hsa04670 | Leukocyte transendothelial migration - Homo sapiens (human) | http://www.genome.jp/kegg-bin/show\_pathway?hsa04670+29119+387+7070+10451 | 2.545370e-02 | 4 | 79 | 112 | 7906 | 1.000000e+00 | 1.594249 | 0.050633 | CTNNA3//RHOA//THY1//VAV3 |
| hsa04392 | Hippo signaling pathway - multiple species - Homo sapiens (human) | http://www.genome.jp/kegg-bin/show\_pathway?hsa04392+4771+7005 | 3.362923e-02 | 2 | 79 | 29 | 7906 | 1.000000e+00 | 1.473283 | 0.025316 | NF2//TEAD3 |
| hsa04520 | Adherens junction - Homo sapiens (human) | http://www.genome.jp/kegg-bin/show\_pathway?hsa04520+10458+29119+387 | 3.378364e-02 | 3 | 79 | 71 | 7906 | 1.000000e+00 | 1.471294 | 0.037975 | BAIAP2//CTNNA3//RHOA |
| hsa00630 | Glyoxylate and dicarboxylate metabolism - Homo sapiens (human) | http://www.genome.jp/kegg-bin/show\_pathway?hsa00630+1431+5096 | 3.580199e-02 | 2 | 79 | 30 | 7906 | 1.000000e+00 | 1.446093 | 0.025316 | CS//PCCB |
| hsa05100 | Bacterial invasion of epithelial cells - Homo sapiens (human) | http://www.genome.jp/kegg-bin/show\_pathway?hsa05100+10094+29119+387 | 3.624998e-02 | 3 | 79 | 73 | 7906 | 1.000000e+00 | 1.440692 | 0.037975 | ARPC3//CTNNA3//RHOA |
| hsa00051 | Fructose and mannose metabolism - Homo sapiens (human) | http://www.genome.jp/kegg-bin/show\_pathway?hsa00051+8789+5209 | 4.263299e-02 | 2 | 79 | 33 | 7906 | 1.000000e+00 | 1.370254 | 0.025316 | FBP2//PFKFB3 |
